# Development of an Instrument to Assess Parents’ Excessive Web-Based Searches for Information Pertaining to Their Children’s Health: The “Children’s Health Internet Research, Parental Inventory” (CHIRPI)

**DOI:** 10.2196/16148

**Published:** 2020-04-15

**Authors:** Antonia Barke, Bettina K Doering

**Affiliations:** 1 Clinical and Biological Psychology Catholic University Eichstaett-Ingolstadt Eichstaett Germany

**Keywords:** hypochondriasis, internet, health behavior, health knowledge, attitudes, practice, questionnaire, children, parents

## Abstract

**Background:**

People often search the internet to obtain health-related information not only for themselves but also for family members and, in particular, their children. However, for a minority of parents, such searches may become excessive and distressing. Little is known about excessive web-based searching by parents for information regarding their children’s health.

**Objective:**

This study aimed to develop and validate an instrument designed to assess parents' web-based health information searching behavior, the Children’s Health Internet Research, Parental Inventory (CHIRPI).

**Methods:**

A pilot survey was used to establish the instrument (21 items). CHIRPI was validated online in a second sample (372/384, 96.9% mothers; mean age 32.7 years, SD 5.8). Item analyses, an exploratory factor analysis (EFA), and correlations with parents’ perception of their children’s health-related vulnerability (Child Vulnerability Scale, CVS), parental health anxiety (modified short Health Anxiety Inventory, mSHAI), and parental cyberchondria (Cyberchondria Severity Scale, CSS-15) were calculated. A subset of participants (n=73) provided retest data after 4 weeks. CHIRPI scores (total scores and subscale scores) of parents with a chronically ill child and parents who perceived their child to be vulnerable (CVS+; CVS>10) were compared with 2×2 analyses of variances (ANOVAs) with the factors Child’s Health Status (chronically ill vs healthy) and perceived vulnerability (CVS+ vs CVS−).

**Results:**

CHIRPI’s internal consistency was standardized alpha=.89. The EFA identified three subscales: Symptom Focus (standardized alpha=.87), Implementing Advice (standardized alpha=.74) and Distress (standardized alpha=.89). The retest reliability of CHIRPI was measured as *r*_tt_=0.78. CHIRPI correlated strongly with CSS-15 (*r*=0.66) and mSHAI (*r*=0.39). The ANOVAs comparing the CHIRPI total score and the subscale scores for parents having a chronically ill child and parents perceiving their child as vulnerable revealed the main effects for perceiving one’s child as vulnerable but not for having a chronically ill child. No interactions were found. This pattern was observed for the CHIRPI total score (η^2^=0.053) and each subscale (Symptom Focus η^2^=0.012; Distress η^2^=0.113; and Implementing Advice η^2^=0.018).

**Conclusions:**

The psychometric properties of CHIRPI are excellent. Correlations with mSHAI and CSS-15 indicate its validity. CHIRPI appears to be differentially sensitive to excessive searches owing to parents perceiving their child’s health to be vulnerable rather than to higher informational needs of parents with chronically ill children. Therefore, it may help to identify parents who search excessively for web-based health information. CHIRPI (and, in particular, the Distress subscale) seems to capture a pattern of factors related to anxious health-related cognitions, emotions, and behaviors of parents, which is also applied to their children.

## Introduction

### Background

The internet is a ubiquitous source of information. In 2017, 3.6 billion people worldwide have used the internet [1]. More than half of them (55%‑85%) used the internet to search for health-related information [2-5]. Seeking health information on the internet has many benefits, such as convenience, greater perceived anonymity regarding sensitive issues [6,7], and health service user empowerment [2,8-10]. However, a variety of downsides have also been described. The quality of many health-related websites has come under scrutiny [9,11-13]. The dangers of using search engines to find health information, eg, escalating searches that lead the user from researching harmless symptoms to self-diagnosing with life-threatening diseases, have been pointed out [12-16]. The users’ ability to appraise the material and filter irrelevant and unreliable information has also been questioned [11,12,16-19]. Some negative consequences of the relationship between health providers and patients have been reported [20,21]. Examples for such consequences comprise patients requesting inappropriate treatments on the basis of inaccurate, or inaccurately understood, Web-based information and frustration on the health professional’s side (due to challenges to their expert opinion and time used to explain) and the patient’s side (due to perceived unresponsiveness of the health professional to the Web-based researches).

People do not just search for information regarding their own health, but they also search for information relevant to the health of others in their family, particularly their children [3,4]. The reported prevalence of such searches varies from 30% to 35% [10,22], over 42% [16], 50% [3,4], and 56% [23] to 75% [24]. Searching for information related to one’s children’s health can be an expression of increased informational needs in the face of the new responsibilities of parenting [25].

Data about parents’ web searches for information related to their children’s health mirror data about Web searches for health information in general. Studies have reported that the majority of people (>60%) searching online for health information used search engines to find the websites [10,18] and that 75% of parents looking for information about their child’s condition used Google [26]. Concerns about the quality of Web-based health information in general are mirrored by concerns about websites regarding children’s health issues [14,27-30] including vaccination [31,32], other procedures [33], specific conditions such as prematurity [34], illnesses such as eczema [35], and more general health advice, such as recommendations for children’s toothbrushing [36].

The reported benefits of searching online for information related to one’s children’s health include being better informed about health and health care provision [37], the availability of information outside doctors’ hours [25], decreased use of services for minor, nonurgent issues [38], and improved decision making in urgent cases [38]. Sometimes parents are even able to arrive at the correct diagnosis when the attending pediatrician has failed to do so [39]. The vast majority (89%‑90%) of parents who have used the internet to find information related to their children’s health found it useful [26,40,41] and many (67%) said it had influenced the medical decisions made on behalf of their child [40]. Interestingly, only a minority of parents chose to discuss the results of their Web search with their child’s doctor [26].

However, despite the clear benefits of searching the internet for health information, 18% [26] to 30% [42] of parents reported finding the websites confusing and distressing or felt more anxious after their search. In other words, in a small number of families, parents’ search for Web-based health information can have detrimental effects. Apart from reports of cases where attempts to implement advice obtained online have gone wrong [27], parents’ searches can negatively affect communication with health professionals [22,42-45] and lead to distress and anxiety for the parents [22,26,42]. In the case of searches for information regarding one’s own health, a similar pattern of behavior, termed “cyberchondria,” has been described [46-51]. Cyberchondria is characterized by Web searches for information about perceived symptoms and possible diagnoses. The searches are undertaken with the aim of assuaging worries and relieving anxiety, yet they increase them and potentially lead to further searches in a quest for reassurance. The increased worry may be a consequence of the search itself, the material encountered online, or the accompanying attentional and cognitive processes. Cyberchondria is a multidimensional construct with five main facets: compulsion, ie, Web searches interrupt activities of daily living; distress, ie, search behavior has negative emotional consequences; excessiveness, ie, repeated searching and, sometimes, escalating search behavior; need for reassurance, ie, desire to consult a health care professional after the Web search; mistrust of medical professionals, ie, putting more faith in the information one has researched by oneself than in trusting expert opinion. Cyberchondria is related to health anxiety [52,53]. The vicious circle that occurs when searching for health information for oneself [46] may also apply when searching for information related to one’s children’s health. Some authors call this pattern of behavior “cyberchondria by proxy” [22]. We do not recommend usage of this term as it suggests a resemblance to the Munchausen syndrome by proxy and thus appears unnecessarily stigmatizing, especially given that hardly anything is known about the consequences of such parental searches. As a neutral phrase, we suggest using “excessive search for Web-based information related to one’s children’s health.”

### Objectives

The aim of the study was to improve knowledge regarding parents’ excessive Web-based searching for information related to one’s children’s health by developing an instrument to capture such search behavior. We also aimed at investigating whether such behavior is associated with personal health anxiety and with searching for Web-based health information related to one’s own health, the perceived health status of one’s child, and other relevant health information, such as a chronic illness of the child or of the parents.

## Methods

### Ethics

The study was conducted according to the Declaration of Helsinki and approved by the Internal Review Board of Göttingen University (number 2017-162). Data were collected anonymously, and participants were not able to access the survey until they had viewed information about the study and provided consent.

### Questionnaire Development

The first step was the generation of an item pool through brainstorming by the researchers and advanced students familiar with the construct of cyberchondria. We reviewed the existing research literature as referenced in the Introduction section. As no instruments exist to assess excessive searching for Web-based information related to one’s children’s health, we especially considered research in the field of adult cyberchondria [47-52] and previously established measurement instruments for cyberchondria such as the Cyberchondria Severity Scale (CSS) [48] as the basis for item development. Items of the CSS were assessed with regard to their suitability for parents’ Web searches, and select item content was adapted for the item pool. In addition, informal interviews were conducted with parents of children aged 0 to 10 years, clinical psychologists specialized in treating children and adolescents, nursery school teachers, and pediatricians. This resulted in a pool of 84 items that were screened to remove items regarded as too linguistically complicated (eg, double negations, uncommon words) or redundant, leaving a final pool of 63 items. The 63 items were presented, in a randomized order, to an online sample of parents of children between the ages of 0 and 10 years. The parents were recruited online through parenting forums and advertisements on notice boards. A total of 486 parents, almost all being mothers (471/486, 96.9%), completed the questionnaire. Their mean age was 31.9 years (SD 5.3). After an analysis of the distribution of missing answers, item difficulty, and participant comments, exploratory factor analyses (EFAs) and reliability analyses were conducted. Items were selected in an iterative process with items excluded if they showed unsatisfactory item characteristics.

The final instrument, *Children’s Health Internet Research Parental Inventory* (CHIRPI), consisted of 21 items organized into three 7-item subscales. The Distress subscale captures the aversive emotional consequences of parents’ Web searches for information related to their children’s health, such as anxiety or irritation, captures physiological arousal, such as difficulty falling asleep or finding it hard to relax, and captures the interference of these consequences with everyday activities. The Symptom Focus subscale captures parents’ search behavior that is prompted by a perceived physical change in their children and is aimed at finding the causes for the symptoms or a cure for a presumed disease. The Implementing Advice subscale captures parents’ tendency to implement Web-based advice and encompasses various behaviors, such as asking the child’s doctor to prescribe specific medication or carry out particular diagnostic procedures.

### Procedure Questionnaire Validation

#### Procedure

For the validation study, a Web-based questionnaire was generated using Lime Survey (Limesurvey GmbH, Hamburg, Germany) [54]. For a complete description according to the Checklist for the Reporting Results of Internet E-Surveys (CHERRIES [55]), see Multimedia Appendix 1. After viewing information about the study and providing consent, participants were linked to a questionnaire, which asked them to provide demographic information about themselves and their children. It included items on thoughts, emotions, and behaviors regarding their children’s health. Then participants completed CHIRPI. This section was followed by questionnaires assessing the perceived health-related vulnerability of their children and parents’ health anxiety and cyberchondria (see the following sections). At the end, participants were invited to take part in a brief retest in about three weeks’ time. If they chose to do so, they were asked to generate a code so that their retest results could be linked with their original results while preserving their anonymity.

#### Material

##### Demographic Information About Parents and Children

The participants answered questions about their own age, sex, education, mother tongue, current employment, and whether they were a single parent. They also reported how many children they had and the age and sex of each child.

##### Health-Related Information

The participants were asked whether they worked in a health-related profession (yes/no), whether they, the other parent, or any of their children suffered from a chronic illness (yes/no). They also used a visual analogue scale (VAS;implemented as a horizontal slider) to respond to the following questions: (1) How anxious/worried are you about your child’s health? (0=not at all anxious/worried to 100=very anxious/worried); (2) How do you rate your own medical knowledge/competence? (0=not at all competent to 100=very competent); (3) How would you rate your child’s health? (0=very poor to 100=very good); (4) How has your experience with your child’s doctors been? (0=very poor to 100=very good).

In addition, the participants reported how much time (in minutes) they spent searching the Web for information related to their children’s health in a typical week in which their child (1) displays symptoms of some kind and (2) does not display any symptoms.

##### Parents’ Searches for Web-Based Information Related to Their Children’s Health

The validation version of CHIRPI consists of 21 items describing the cognitive, emotional, and behavioral aspects of parents’ Web searches for information about their children’s health (see the previous sections). Participants used a Likert scale (1=never to 5=always) to indicate how often they exhibited the behavior or experienced the emotional or cognitive state described. See Multimedia Appendix 2 for the questionnaire in German and English.

##### Perceived Health-Related Vulnerability of the Child or Children

The parents’ perception of the child’s health-related vulnerability was measured with the *Child Vulnerability Scale* (CVS) [56]. As no German version existed, we translated the CVS according to established guidelines for such translations [57]. The scale consists of eight items describing parental perceptions of the child’s health (eg, “In general my child seems less healthy than other children” and “When something is going around, my child usually catches it.”). Parents responded using a 4-point scale (0=strongly disagree to 3=strongly agree). Scores can range from 0 to 24, with higher scores representing greater perceived vulnerability. Scores of 10 or more indicate that the parent perceives his or her child’s health to be vulnerable [56]. The CVS had an internal consistency of Cronbach alpha=.74 in the original study [56] and alpha=.80 in our sample.

##### Anxiety Regarding the Parents’ Own Health

Parents’ anxiety about their own health was assessed with the modified version of the *Short Health Anxiety Inventory* (mSHAI) [58]. The mSHAI uses simpler answer formats than the original [59]; it consists of 14 items on health anxiety (eg, “I spend much of my time worrying about my health”), to which responses are given using a 5-point scale (1=strongly disagree to 5=strongly agree). Higher scores indicate greater health anxiety [60]. In our sample, the mSHAI had an internal consistency of standardized alpha=.94.

##### Cyberchondria With Regard to One’s Own Health

Parents’ tendency to conduct Web searches for information relating to their own health and the negative consequences of such searches were measured with the short version of the *Cyberchondria Severity Scale-15* (CSS-15) [46,48]. The CSS-15 consists of 15 items describing possible cognitions, emotions, or behaviors related to Web searches for health-related information (eg, “I feel more anxious or distressed after researching symptoms or perceived medical conditions online.”) to which responses are given using a 5-point scale (1=never to 5=always). The short version showed good validity and good internal consistency in the original study (standardized alpha=.82) [46] and in our sample (standardized alpha=.84).

### Retest

Participants who agreed to take part in a retest received an email invitation with a link to a short version of the Web-based survey 3 weeks after they had first completed it. They repeated basic demographic information (described in the Demographic Information About Parents and Children section), health-related information (described in the Health-Related Information section), and CHIRPI.

### Data Analysis

We conducted a standard item analysis for CHIRPI, first inspecting the number of missing data points for each item and calculating item means, standard deviations, and difficulties. Interitem correlations and item-total correlations were also computed.

We investigated the factorial structure of CHIRPI using an EFA. The suitability of the data for a factor analysis was ascertained using the Kaiser-Meyer-Olkin (KMO) criterion and Bartlett’s test. The number of factors to be extracted was determined using a Velicer minimum average partial (MAP) test [61]. Factors were extracted using a maximum likelihood estimation, and the extracted factors were rotated obliquely (Promax with a Kaiser normalization).

Reliability analyses were carried out by computing the internal consistency (standardized alpha) of the whole scale and the subscales and examining whether the scale and subscales would have benefitted from the removal of individual items. The retest reliabilities of the whole scale and the subscales were calculated as Pearson correlations between the first and second measurements. Parents who completed the retest too late (more than 5 weeks after first completing CHIRPI) were excluded from the retest. We also used independent sample *t* tests (2-tailed) to determine whether the subsample that had agreed to take part in the retest differed from the subsample that chose not to participate with regard to age, number of children, time spent searching for Web-based information related to their children’s health, and scores in CHIRPI, CSS-15, mSHAI, and CVS.

The correlations were calculated between the CHIRPI scale and its subscales and the demographic information, global (VAS) ratings regarding worry about the child’s health, perceived parental medical competence, perceived general health of the child, and previous experience with the child’s doctors. In addition, we calculated the correlations between CHIRPI scores and scores in CVS, mSHAI, and CSS-15.

We used a 2×2 analysis of variance (ANOVA) with the factors child’s health status (healthy/chronically ill) and the perceived health vulnerability of the child (CVS−: CVS below cutoff/CVS+: CVS above cutoff), and the CHIRPI total score as a dependent variable to investigate the influence of the child’s objective health status and the parent’s perceptions of the child’s vulnerability on the CHIRPI score. These analysis was repeated for the subscales. As a measure of effect size, we reported eta-squared [62]. The data were analyzed using Statistica (TIBCO Software Inc, 2018, version 13, Palo Alto, CA), except for the EFA, which was conducted using SPSS (IBM, version 25, Armonk, New York).

### Participants

The Web-based survey was widely publicized on German webpages and social media related to parenthood and children. Participation was open to all interested parents, and the resulting sample constituted a convenience sample. Participants were included in the analysis if they reported German as their native language and had at least one child under 10 years of age. A total of 515 participants gave informed consent, and 491 of the participants provided the basic data required to assess eligibility. The inclusion criteria were fulfilled by 480 respondents (nine reported another native language and in two cases the youngest child was older than 10 years). A total of 92 of the 480 respondents (92/480, 19.2%) stopped responding before they had completed CHIRPI. Furthermore, two participants provided implausible data and had to be excluded.

Data from a final set of 384 participants (372/384, 96.9% mothers and 12/384, 3.1% fathers) were analyzed. The mean age of the sample was 32.7 years (SD 5.8) and they had an average of 1.8 children (186 participants had 1 child, 137 participants had 2 children, 40 participants had 3 children, 13 participants had 4 children and 8 participants had five or more children). The mean age of the participants’ youngest child was 3.4 years (SD 3.0). Approximately one in 10 (37/384, 9.6%) of the participating parents reported being single parents and a quarter (94/384, 25.4%) worked in a health-related profession. In total, 11.7% (45/384) reported that at least one of their children had a chronic illness, 22.9% (88/384) said they personally had a chronic illness, and 15.9% (61/384) said their partner suffered from a chronic illness; 37.8% (145/384) reported that at least one member of their immediate family had some form of a chronic illness.

## Results

### Item Analysis

There were very few missing responses in CHIRPI. The frequency of missing responses ranged from 0 (most items) to 3 (item 20; see Table 1). Item 16 was the most difficult, ie, least endorsed (p_i_=0.07) and item 2, the easiest, ie, most endorsed (p_i_=0.65). The mean item difficulty was p_i_=0.29, and the interitem correlation for the whole scale was *r*=0.29. Item-total correlations ranged from *r*_itc_=0.37 (item 20) to *r*_itc_=0.65 (item 10), with a mean of *r*_itc_=0.50.

**Table 1:** Item means and standard deviations, missing responses per item, item difficulties, item-total correlations, and the internal consistency (standardized alpha) of the scale with single item exclusions (n=384).

Item	Value, mean (SD)	Missing responses, n	Difficulty (p_i_)	Item-total correlation (r_itc_)	Standardized alpha (if item is excluded)
1	3.2 (1.01)	0	0.56	0.52	.89
2	3.6 (1.08)	0	0.65	0.51	.89
3	1.8 (0.89)	0	0.20	0.38	.89
4	2.3 (0.92)	1	0.33	0.56	.89
5	1.5 (0.80)	0	0.11	0.38	.89
6	2.3 (0.96)	0	0.32	0.56	.89
7	1.7 (0.94)	0	0.17	0.55	.89
8	2.4 (0.85)	0	0.34	0.52	.89
9	2.0 (0.95)	0	0.25	0.58	.89
10	3.0 (1.03)	2	0.51	0.65	.89
11	1.7 (0.90)	0	0.17	0.40	.89
12	1.6 (0.84)	0	0.14	0.50	.89
13	1.7 (0.85)	1	0.16	0.43	.89
14	3.1 (1.11)	1	0.52	0.55	.89
15	1.6 (0.85)	1	0.15	0.53	.89
16	1.3 (0.60)	1	0.07	0.43	.89
17	2.8 (1.17)	0	0.45	0.56	.89
18	1.4 (0.71)	0	0.09	0.48	.89
19	3.3 (1.17)	0	0.57	0.58	.89
20	1.8 (0.89)	3	0.21	0.37	.89
21	1.7 (0.91)	0	0.19	0.58	.89

### Factor Structure

The KMO criterion value of 0.88 and the Bartlett test chi-square value of 3421.3 (*P*<.001) indicated that the data were suitable for an EFA. The Velicer MAP test suggested the extraction of three factors. The maximum likelihood factor estimation converged after six iterations and three factors were extracted, which together explained 45.16% of the variance in the CHIRPI scores. The factor loadings ranged from 0.42 to 0.86, and no item showed double loadings of >0.30 with another factor (Table 2). Factor 1 aggregated items that were indicative of distress as a consequence of searching and was called “Distress.” Factor 2 was related to a symptom-focused search strategy and was named “Symptom Focus.” Factor 3 encompassed various behaviors aimed at implementing advice found online and was called “Implementing Advice.” As the factors showed good internal consistency, we treated them as subscales. The subscales were moderately correlated, Distress×Symptom Focus: *r*=0.36 (*P*<.001); Distress×Implementing Advice: *r*=0.44 (*P*<.001); Symptom Focus×Implementing Advice: *r*=0.50 (*P*<.001).

**Table 2:** Results of the exploratory factor analysis: factor loadings, eigenvalues, and percentage of variance explained.

Item^a^	Factor 1: Distress^b^	Factor 2: Symptom focus^c^	Factor 3: Implementing advice^d^
15. I have difficulty falling asleep after searching online for information about illnesses or symptoms concerning my child.	0.87	−0.08	−0.02
21. I find it difficult to stop worrying about my child’s health after searching online for information about illnesses or symptoms concerning my child.	0.86	−0.02	−0.02
9. After I have searched online for information about illnesses or symptoms that concern my child, I feel more anxious and distressed than before.	0.80	0.06	−0.07
7. I panic when I read online that one of my child’s symptoms occurs in a rare or serious disease.	0.67	0.16	−0.10
4. I find it hard to relax after searching online for health information concerning my child.	0.67	−0.10	0.15
12. I become angry and irritated more easily after reading about my child’s symptoms or illnesses online.	0.62	0.13	0.00
18. Searching online for health information relevant to my child interferes with my everyday activities, such as chores, hobbies or spending time with family and friends.	0.50	−0.09	0.27
10. When my child displays any symptom, I search on the Internet for information about it.	−0.03	0.80	0.05
17. When searching online for health information relevant to my child, I am mainly interested in what illness the observed symptoms point to.	0.17	0.76	−0.19
1. When I notice any change in my child’s body I search for information about it on the Internet.	−0.06	0.73	−0.03
2. When I search online for health information relevant to my child, I am particularly interested in whether the problems or symptoms need treatment.	−0.07	0.68	0.02
6. Based on my online searches for health information relevant to my child's symptoms, I suspect he or she has a particular disease.	0.06	0.66	−0.03
19. When searching online for health information relevant to my child I am hoping to find practical advice.	−0.03	0.59	0.20
14. When searching online for health information relevant to my child, I am particularly interested in possible causes for the illness.	0.06	0.45	0.21
5. After I have searched online for health information I ask teachers or nursery staff for help in implementing the recommendations.	−0.01	−0.14	0.68
3. After I have searched online for health information I have prescribed certain activities to my child (e.g. exercise for muscle strengthening)	−0.08	0.08	0.52
16. I ask my child’s doctor to prescribe particular drugs that I have read about online.	0.12	−0.04	0.50
8. When I find advice or recommendations during my online searches for health information (e.g. a particular diet) I apply them to my child.	0.11	−0.06	0.49
11. After searching online for health information I ask teachers or nursery staff for their help in observing my child’s symptoms.	−0.04	0.25	0.48
20. If I see freely available medication recommended during my online search for health information, I will give that medication to my child.	−0.07	0.16	0.43
13. I suggest specific diagnostic investigations that I have read about online to my child’s doctor.	0.08	0.09	0.42

^a^Factor extraction method: maximum likelihood estimation; rotation: Promax with a Kaiser normalization.

^b^Eigenvalue=6.24; explained variance=29.71%; cumulative explained variance=29.71%.

^c^Eigenvalue=2.14; explained variance=10.21%; cumulative explained variance=39.92%.

^d^Eigenvalue=1.10; explained variance=5.24%; cumulative explained variance=45.16%.

### Reliability

#### Internal Consistency

The whole scale had an internal consistency of standardized alpha=.89 and would not have been improved by the exclusion of any item. The internal consistencies of the subscales were as follows: Symptom Focus alpha=.87, Implementing Advice alpha=.74, and Distress alpha=.89 (Table 3). None of the subscales would have benefitted from excluding an item.

**Table 3:** Means, standard deviations, interitem correlations, mean item-total correlations, standardized alpha, and test-retest reliability of the Children’s Health Internet Research Parental Inventory (CHIRPI) and its subscales.

Scale	Value, mean (SD)	Mean interitem correlation	Mean item-total correlation, *r*_itc_	Standardized alpha	Test-retest reliability, *r*_tt_
Distress	12.3 (4.77)	0.54	0.68	.89	0.84
Symptom focus	21.4 (5.59)	0.48	0.64	.87	0.70
Implementing advice	12.0 (3.57)	0.29	0.45	.74	0.61
CHIRPI (total scale)	45.7 (11.00)	0.29	0.50	.89	0.78

#### Retest Reliability

We calculated the 4-week-retest reliability of the subscales and the whole scale. A total of 96 participants agreed to take part in the retest and provided pseudonyms that could be matched with those provided in the original survey; 23 of them were excluded because they were late to fill in the retest. In the end, 73 datasets were included in the test-retest analysis, with a mean test-retest interval of 27.9 days (SD 3.1; range: 21‑38). The retest reliabilities were as follows: *r*_tt_=0.78; *P*<.001 (whole scale); *r*_tt_=0.67; *P*<.001 (Symptom Focus), *r*_tt_=0.59; *P*<.001 (Implementing Advice), and *r*_tt_=0.84; *P*<.001 (Distress). The retest subsample did not differ from participants not included in the retest with regard to age, sex, or any questionnaire score but, on average, they had fewer children (for the full results, see Multimedia Appendix 3).

### Indicators of Validity

#### Correlations With Health-Related Measures and Other Variables

The CHIRPI score was uncorrelated with parents’ age and the level of education. Weak negative correlations indicated that parents with older children or more children tended to score lower on CHIRPI. There were moderate positive correlations between the CHIRPI score and time spent searching for Web-based health information in weeks when the child displayed symptoms (*r*=0.26) and a small correlation in symptom-free weeks (*r*=0.14). There was also a moderate correlation (*r*=0.38) between the CHIRPI score and the VAS rating of worry and anxiety about one’s child’s health. This correlation was clearly related to the greater distress that these parents felt regarding their search. In addition, there was a small (*r=*−0.20) negative association showing that the poorer the participants rated their experience with the child’s doctors, the greater were their scores on CHIRPI. By far the strongest correlation was observed between the CHIRPI score and parental cyberchondria (*r*=0.66) followed by personal health anxiety of the parents (*r*=0.39) and parents’ perception of the vulnerability of their child’s health (*r*=0.29; see Table 4).

Turning to the subscales, the highest correlations involved Distress, which was strongly correlated with the CSS-15 (*r*=0.61), the mSHAI (*r*=0.50), the VAS rating of worry and anxiety regarding the child’s health (*r*=0.50), and the perceived vulnerability regarding one’s child (*r*=0.54). The other subscales were also most highly correlated with the CSS-15, *r*=0.51 (Symptom Focus) and *r*=0.46 (Implementing Advice). Details of all correlations are given in Table 4.

**Table 4:** Correlations between the full scale or subscales and demographic variables, time spent searching the internet for information related to one’s children’s health, health-related questionnaires, and the visual analog scales.

Variables^a^	Value, mean (SD)	CHIRPI^b^ score		Distress		Symptom focus		Implementing advice	
		*r*	*P* value	*r*	*P* value	*r*	*P* value	*r*	*P* value
Age	32.7 (5.8)	−0.01	.88	−0.04	.47	0.02	.69	−0.03	.61
Years of education	16.2 (4.1)	0.00	.93	−0.07	.17	0.10	.046	−0.12	.02
Number of children	1.8 (1.1)	−0.12	.02	−0.09	.07	−0.15	.002	0.00	.96
Mean age of children (years)	3.4 (3.0)	−0.17^a^	<.001	−0.06	.24	−0.27^a^	<.001	0.03	.56
Search time, week without symptoms (mins)	7.9 (24.0)	0.14	.007	0.04	.49	0.11	.03	0.19^a^	<.001
Search time, week with symptoms (mins)	39.8 (52.5)	0.26^a^	<.001	0.11	.04	0.27^a^	<.001	0.20^a^	<.001
CVS^c^	5.9 (4.3)	0.29^a^	<.001	0.54^a^	<.001	0.15^a^	.005	0.27^a^	<.001
mSHAI^d^	27.3 (11.1)	0.39^a^	<.001	0.50^a^	<.001	0.21^a^	<.001	0.22^a^	<.001
CSS-15^e^	29.4 (8.0)	0.66^a^	<.001	0.61^a^	<.001	0.51^a^	<.001	0.46^a^	<.001
VAS^f^ worry/anxiety regarding child’s health	49.3 (26.4)	0.38^a^	<.001	0.50^a^	<.001	0.22^a^	<.001	0.21^a^	<.001
VAS child’s health	86.5 (15.4)	−0.09	.08	−0.11	.03	0.00	.97	−0.09	.095
VAS participant’s medical competence	61.9 (22.3)	0.03	.64	−0.08	.18	0.02	.68	0.14	.015
VAS experience with child’s doctors	75.6 (23.6)	−0.20^c^	<.001	−0.09	.11	−0.19	<.001	−0.19^a^	<.001

^a^Significant after Bonferroni correction.

^b^CHIRPI: Children’s Health Internet Research Parental Inventory.

^c^CVS: Child Vulnerability Scale.

^d^mSHAI: Modified Health Anxiety Inventory.

^e^CSS-15: Cyberchondria Severity Scale (15-item version).

^f^VAS: visual analog scale (0‑100; 0 signifying no worries about the child’s health, child’s health is poor, parent has no medical competence, parent’s experience with child’s doctors has been poor).

#### Particular Groups of Parents

We identified groups of parents that may have different attitudes toward Web-based searches for health information regarding their children. The first group comprised parents with a chronically ill child (as opposed to parents of healthy children). The second group consisted of parents who perceived their child as being vulnerable with regard to health (CVS+ as opposed to CVS−). Within both groups, we compared the parents with their counterparts regarding the search behavior, health anxiety, and CHIRPI scores.

#### Parents With Chronically Ill Children

In our sample, 11.7% (45/384) of parents reported that they had a child with a chronic illness. They spent about twice as much time searching for Web-based health information in weeks with symptoms (mean 62.4 min, SD 95.6) compared with parents with healthy children (mean 36.9 min, SD 43.5) and worried more about their child’s health (mean 60.9 min, SD 22.8) than their counterparts (mean 48.1 min, SD 26.5). Despite this, the only CHIRPI subscale on which they scored higher than parents of healthy children was Implementing Advice (mean 13.5, SD 4.4 vs mean 11.9, SD 3.6; see Table 5).

**Table 5:** Comparisons of parents with chronically ill children and parents with healthy children by means of independent samples t tests.

Variable		Chronically ill child (n=45), mean (SD)	Healthy child (n=337), mean (SD)	*t* value (df)	*P* value	Cohen *d*
**CHIRPI^a^**		48.9 (13.7)	45.2 (11.0)	2.04 (380)	.04	0.30
	Distress	13.6 (5.8)	12.2 (4.8)	1.74 (380)	.08	0.26
	Symptom focus	21.9 (5.6)	21.3 (5.7)	0.67 (380)	.50	0.11
	Implementing advice	13.5 (4.4)	11.9 (3.6)	2.62 (380)	.009	0.38
CVS^b^		7.7 (4.5)	5.7 (4.2)	2.93 (351)	.004	0.47
mSHAI^c^		27.7 (9.5)	27.2 (11.4)	0.25 (345)	.81	0.04
CSS-15^d^		30.2 (8.0)	29.3 (8.0)	0.67 (335)	.50	0.11
Search time, week without symptoms (minutes)		14.7 (30.5)	7.1 (23.0)	1.93 (373)	.05	0.28
Search time, week with symptoms (minutes)		64.2 (95.6)	36.9 (43.5)	3.24 (378)	.001	0.37
VAS^e^ worry/anxiety regarding child’s health		60.9 (22.8)	48.1 (26.5)	2.78 (335)	.006	0.52
VAS child’s health		73.8 (20.2)	88.2 (13.9)	−5.83 (361)	<.001	−0.83
VAS participant’s medical competence		72.7 (19.1)	60.6 (22.3)	3.08 (307)	.002	0.59
VAS experience with child’s doctors		63.8 (33.4)	77.1 (21.7)	−3.23 (321)	.001	−0.47

^a^CHIRPI: Children’s Health Internet Research Parental Inventory.

^b^CVS: Child Vulnerability Scale.

^c^mSHAI: Modified Health Anxiety Inventory.

^d^CSS-15: Cyberchondria Severity Scale (15-item version).

^e^VAS: visual analog scale (0-100 with 0 designating no worry regarding child’s health, poor child’s health, no medical competence, poor experiences with child’s doctors).

#### Perceived Health-Related Vulnerability of the Child

We compared parents who perceived their child’s health to be vulnerable with parents who perceived their child’s health to be less vulnerable. CVS+ parents reported markedly higher scores in CHIRPI (mean 52.0, SD 13.6) than their CVS− counterparts (mean 44.0, SD 10.1) and scored particularly high on the Distress subscale (CVS+: mean 16.4, SD 6.3; CVS−: mean 11.3, SD 4.0; Table 6). They also reported higher personal health anxiety (CVS+: mean 35.7, SD 11.9; CVS−: mean 25.3, SD 10.0), higher cyberchondria related to their own health (CVS+: mean 34.1, SD 9.6; CVS−: mean 28.3, SD 7.1), and greater worry and anxiety about their child’s health (CVS+: mean 65.9, SD 22.0; CVS−: mean 44.9, SD 25.7) but did not report spending more time on Web searches. See Table 6 for the full results.

**Table 6:** Comparison of parents above (CVS+) and below (CVS−) the cutoff for the Child Vulnerability Scale using independent samples t tests.

Variable		CVS−^a^ (n=283), mean (SD)	CVS+ (n=72), mean (SD)	*t* test (df)	*P* value	Cohen *d*
**CHIRPI^b^**		52.0 (13.6)	44.0 (10.1)	5.52 (353)	<.001	0.66
	Distress	16.4 (6.3)	11.3 (4.0)	8.55 (353)	<.001	0.98
	Symptom Focus	22.9 (5.8)	20.9 (5.5)	2.69 (353)	<.001	0.35
	Implementing Advice	13.5 (4.3)	11.8 (3.4)	3.57 (353)	<.001	0.44
mSHAI^c^		35.7 (11.9)	25.3 (10.0)	7.42 (347)	<.001	0.95
CSS-15^d^		34.1 (9.6)	28.3 (7.1)	5.56 (337)	<.001	0.69
Search time, week without symptoms (minutes)		6.9 (12.6)	8.7 (27.0)	−0.52 (347)	.60	−0.08
Search time, week with symptoms (minutes)		44.4 (43.0)	40.4 (56.1)	0.56 (351)	.58	0.08
VAS^e^ worry/anxiety regarding child’s health		65.9 (22.0)	44.9 (25.7)	−6.1 (311)	<.001	0.88
VAS child’s health		78.1 (20.5)	88.7 (12.8)	−5.29 (336)	<.001	−0.62
VAS participant’s medical competence		60.0 (24.9)	63.1 (21.3)	−0.95 (291)	.34	-0.13
VAS experience with child’s doctors		69.2 (26.5)	78.1 (21.8)	−2.77 (302)	<.001	−0.37

^a^CVS: Child Vulnerability Scale.

^b^CHIRPI: Children’s Health Internet Research Parental Inventory.

^c^mSHAI: Modified Health Anxiety Inventory.

^d^CSS-15: Cyberchondria Severity Scale (15-item version).

^e^VAS: visual analog scale (0-100; 0 signifying no worries about child’s health, child’s health is poor, parent has no medical competence, parent’s experience of child’s doctors has been poor).

#### Influence of the Child’s Health Status and Parents’ Perception of Their Child’s Health Vulnerability on the CHIRPI Scores

We investigated the relative influence of having a chronically ill child or perceiving the child as vulnerable with regard to their health on CHIRPI and its subscales by calculating four 2×2 ANOVAs with the factors child's health status (healthy/chronically ill) and perceiving the child as vulnerable (CVS−/CVS+). All analyses (CHIRPI total score and each subscale individually) revealed a main effect for the perceived vulnerability but no main effect for the child’s health status or interactions of health status and perceived vulnerability of the child. See Multimedia Appendix 4 and Table 7.

**Table 7:** Results of the 2×2 analyses of variance with the factors child’s health status and perceived vulnerability of the child and the dependent variables, Children’s Health Internet Research, Parental Inventory (CHIRPI) total score, and its subscales.

Analyses of variance (2x2)		Sum of squares	*F* value (df)	*P* value	η^2^
**CHIRPI total score**					
	Intercept	292187.2	2444.256 (1)	N/A^a^	N/A
	Health status	353.0	2.953 (1)	.09	0.008
	Perceived vulnerability	2350.6	19.663 (1)	<.001	0.053
	Health status×perceived vulnerability	77.7	0.650 (1)	.42	0.002
	Error	41719.6	349 (N/A)	N/A	N/A
**CHIRPI Symptom Focus**					
	Intercept	58975.60	1866.339 (1)	N/A	N/A
	Health Status	7.76	0.246 (1)	.62	0.001
	Perceived vulnerability	136.25	4.312 (1)	.04	0.012
	Health status×perceived vulnerability	2.33	0.074 (1)	.79	0.000
	Error	11028.27	349 (N/A)	N/A	N/A
**CHIRPI Distress**					
	Intercept	24622.81	1190.417 (1)	N/A	N/A
	Health status	44.88	2.170 (1)	.14	0.006
	Perceived vulnerability	923.63	44.654 (1)	<.001	0.113
	Health status×perceived vulnerability	15.40	0.745 (1)	.39	0.002
	Error	7218.79	349 (N/A)	N/A	N/A
**CHIRPI Implementing Advice**					
	Intercept	20614.99	1573.060 (1)	N/A	N/A
	Health status	42.28	3.227 (1)	.07	0.009
	Perceived vulnerability	85.44	6.519 (1)	.011	0.018
	Health status×perceived vulnerability	0.29	0.022 (1)	.88	0.000

^a^N/A: not applicable.

## Discussion

### Principal Findings

CHIRPI is the first instrument to assess parents’ excessive Web-based searching for information related to their children’s health. The development of the inventory was guided by previous research on excessive Web-based research for health information [48-52], participative interviews with parents and children’s health professionals, and psychometric standards. The inventory was validated in a good-sized online sample consisting mostly of mothers. Acceptance of the scale was high, and its psychometric characteristics are good to excellent.

### Item Quality

Regarding the quality of the individual items, the general feedback in the validation and pilot tests indicated that the items were understandable and well accepted. The extremely low number of missing responses to individual items also suggested that participants did not find CHIRPI hard to complete. The statistical investigation showed that the item difficulties and item-total correlations were good to very good. In the context of the measurement of attitudes and behaviors, item difficulty is a measure of how frequently the statement made in an item is endorsed. Endorsement rates below 0.20 indicate comparatively low agreement, whereas endorsements above 0.80 point to widespread agreement. The endorsement rates for CHIRPI items ranged from 0.07 (“I ask my child’s doctor to prescribe particular drugs that I have read about online.”) to 0.65 (“When I search online for health information relevant to my child, I am particularly interested in whether the problems or symptoms need treatment.”), with a mean of 0.29. For maximum diagnostic value, item difficulties should range from 0.20 to 0.80. However, considering that we are aiming to assess a behavioral excess, ie, a behavior that is relatively uncommon, the scale should differentiate better in the area of low endorsement. Therefore, the range of difficulties in CHIRPI seems to be appropriate. Item-total correlations capture the relationship between an individual item and the scale as a whole and those of the CHIRPI ranged from 0.37 to 0.65, with a mean of 0.50. These values are medium to high; in general, item-total correlations of 0.30 to 0.50 are regarded as medium and correlations of 0.50 and above, as high.

### Factor Structure

The factor analysis revealed a clear factor structure without any double loadings, consisting of 3 moderately correlated factors, which together explained 45% of the variance. The first factor, Distress, captures how distressed a parent feels as a result of Web searches for health information. It captures cognitive (worry; item 21), emotional (feeling anxious, panicky, angry, or irritated; items 7, 9, and 12), and behavioral (finding it hard to relax or sleep or interference of the search with daily activities; items 4, 15, and 18) aspects of distress. The second factor, Symptom Focus, relates to the characteristics of the search behavior. It captures search behavior focused on changes the parent has noticed in his or her child’s body and on attempts to determine the assumed causes of the symptoms or the presumed underlying illness. The third subscale, Implementing Advice, aggregates behaviors such as following general health advice for one’s child that one has read about online. Examples for such use of internet advice may be the implementation of diets or exercise regimes, administering nonprescription medicine, asking the doctor for prescription drugs or diagnostic procedures, and engaging teachers or the nursery staff’s assistance with observing symptoms or implementing health regimens.

It is important to note both similarities and differences of CHIRPI to the best-established instrument for assessing cyberchondria in adults searching for Web-based health information for themselves [48], especially as CSS-related research informed the generation of CHIRPI. Similar to the CSS, CHIRPI encompasses a Distress Scale, which consists of adapted item content of the respective CSS for parents’ Web searches. It seems that this factor, reflecting the distress after searches, plays an important role in both constructs. In contrast to the CSS, however, CHIRPI hardly contains references to the compulsive nature of the Web searches or the mistrust toward medical professionals. Although items pertaining to this content were part of the original item pool for CHIRPI, they did not perform well during the questionnaire development phase and were thus eliminated by the iterative EFA process—perhaps because these facets are of less importance to parents’ health-related Web searches. Excessiveness of the searches and reassurance by these searches were subsumed under the CHIRPI subscales, Symptom Focus, and Implementing Advice.

### Reliability

We investigated two aspects of reliability: internal consistency and the 4-week retest reliability. The internal consistency of the total scale was high, with standardized alpha=.89, especially given its modest length (21 items). The subscales also showed good (Distress alpha=.89; Symptom Focus alpha=.87) or satisfactory (Implementing Advice alpha=.74) internal consistency. The comparatively low internal consistency of the Implementing Advice subscale might reflect the greater heterogeneity of behaviors described, which is indicated by the lower interitem correlations. Whereas the items of the Distress subscale capture aspects of distress that often co-occur (eg, increased worry, finding it harder to relax, problems with sleep), the items that form the Implementing Advice subscale describe behaviors that—albeit all indicative of the tendency to act on Web-based advice—need not co-occur. Participants who ask nursery staff or teachers for help with observing symptoms may not also ask their child’s doctor to prescribe medications they learned about online.

Test-retest reliability is a measure of the degree of correspondence between questionnaire responses given at different points in time and is influenced by the quality of the questionnaire on the one hand and the stability of the measured construct on the other. At present, little is known about the temporal stability of health-related Web-based search behavior regarding one’s children, in general, or excessive searching for such information, in particular. The 4-week retest reliability of *r*_tt_=0.78 of CHIRPI suggests the phenomenon is stable across a medium time-frame of weeks and indicates that the instrument has good test-retest reliability. The test-retest reliability was highest for the Distress subscale (*r*_tt_=0.84), which suggests that the tendency to experience worry and distress as a consequence of the searching may be an enduring trait. However, although the good test-retest reliability suggests the construct is reasonably stable and that the instrument used to measure it has adequate psychometric characteristics, further research in both these areas is warranted. In particular, it would be instructive to investigate the sensitivity of CHIRPI to change—eg, after an intervention aimed at reducing excessive Web searches by parents. In studies such as ours, where the subsample for the retest is self-selecting, the retest sample may be biased (eg, toward respondents particularly affected by the phenomenon under investigation), so we compared the data of the participants that formed our retest subsample against those who did not provide data for the retest. The samples did not differ with regard to the CHIRPI scores or scores on any of the other questionnaires, but parents with more children were less likely to participate again (small effect), possibly reflecting greater competition for their time.

### Indicators of Validity

We investigated relationships between CHIRPI and related constructs, such as parental cyberchondria and worry about one’s own health and the health of one’s children to provide insights into the instrument’s validity. In addition, we investigated, how the inventory performed in particular groups of parents, namely parents who reported that one of their children had a chronic illness and parents who perceived their child’s health to be vulnerable (based on the CVS score).

#### Correlations With Health-Related Measures and Other Variables

Of all the subscales, the Distress subscale had the highest internal consistency, the highest retest reliability, and the highest correlation with the perceived vulnerability of the child’s health. The Distress score was also highly correlated (*r*=0.46) with the parent’s global rating of his or her worry and anxiety regarding the child’s health. Interestingly, the Distress subscale was the only subscale to show this correlation, indicating that this subscale does assess the specific, distressing aspects of such health concerns. The Distress subscale was most highly correlated with general health anxiety and cyberchondria, ie, excessive Web searches related to one’s own health. This points to the existence of a general pattern of health anxiety-related cognitions, emotions, and behaviors that applies to the health of one’s children as well as to one’s own health

The Symptom Focus subscale was most strongly correlated with parents’ search behavior regarding their own health, as measured by the CSS-15, and with time spent searching in a typical week in which the parent perceives symptoms of some sort in the child. This subscale is the only one that was correlated to the age and number of children. Having more children or older children was associated with a lower Symptom Focus score, although the effects were small to medium. The number of children one has and their mean age are not independent, so the observed effect may reflect a greater perceived need for information on a range of matters (including health) among first-time parents, who are adapting to the new task of parenting [24]. However, given the correlational nature of the data, questions of causality cannot be decided.

The Implementing Advice subscale was moderately correlated with parental cyberchondria and, interestingly, also with negative experiences with the child’s doctors. Regarding the latter association, it is as easy to imagine that dissatisfaction with their child’s doctor would lead parents to look online for advice and to follow such advice as it is to imagine that the parent-physician relationship might be disrupted by parental demands arising from Web-based research.

#### Differentiating the Influence of the Child’s Health Status and Parents’ Perception on the CHIRPI Scores

It seems highly likely that parents whose child has a chronic illness have a heightened need for medical information and make greater use of the internet as a source of such information. In our sample, 11.7% of participants reported that one of their children had a chronic illness, which accords well with the data in a study by Hölling et al [63] who estimated that every eighth child in Germany suffers from some form of chronic illness. This suggests that parents with chronically ill children were neither over- nor under-represented in our sample. Analyzing the differences in health-related Web searches, it appears that, compared with the parents of healthy children, parents with chronically ill children spend about twice as much time searching the internet for information related to their child’s health in weeks in which the child displays symptoms (*d*=0.37). In weeks in which the child does not show any symptoms, no differences between these two groups were found. Parents with chronically ill children also had higher total CHIRPI scores, which could be traced back to the scores on the Implementing Advice subscale (*d*=0.38). However, these two groups of parents did not differ with regard to personal health anxiety and cyberchondria.

The above comparison pattern contrasts with the pattern of comparisons between parents who perceive their child’s health to be vulnerable and those who do not. The former had much higher scores on all CHIRPI scales, as well as on the measures of personal health anxiety and cyberchondria.

To disentangle the contributions to excessive parental search behavior, we calculated an ANOVA that directly compared the influence of the two factors (having a chronically ill child and perceiving one’s child as vulnerable) on CHIRPI scores. We found that CHIRPI is selectively sensitive to the concerns of parents who perceive their children to be vulnerable with regard to health and who show elevated scores of general health anxiety. This may reflect an overarching tendency to worry about health that extends to one’s children. One could speculate that the behavior of parents with a chronically ill child represents a strategy for coping following receipt of a doctor’s diagnosis of a chronic illness in their child. Whereas among parents who perceive their child’s health to be vulnerable, checking them for symptoms, searching on the internet for symptom-related information, and experiencing distress could stem from the same health anxiety that causes these parents to worry about their own health and search the Web for related information. The differences warrant further investigation as such behaviors may relate to the transmission of health anxiety from parents to children. CHIRPI is intended to be sensitive to health anxiety and cyberchondria rather than to an increased need for health information due to chronic medical conditions. Thus, it speaks for the differential validity of CHIRPI and its sensitivity to health anxiety and cyberchondria that it does not show elevated scores for parents with chronically ill children simply in reaction to their legitimate need for health information.

### Limitations

When interpreting the results, the following limitations must be borne in mind. First, our sample consisted predominantly of mothers; however, reflecting offline behaviors [64,65], the majority of those searching online for health information are women [2,10,18,66-68]. Second, our data are cross-sectional, so we cannot draw inferences about causality. Third, all our data are self-reported and may have been affected by a self-selection bias.

### Conclusions

Most parents search on the internet for information related to their children’s health and use it to their advantage. However, for a minority of parents, searching may escalate and have detrimental consequences similar to self-directed cyberchondria. Health professionals working with children worry that parents’ Web searches for health information may affect their own role and their relationships with parents and children [69]. CHIRPI allows us to investigate such issues as it captures various forms of parents’ excessive search behavior (eg, symptom-focused search) and their consequences, such as distress after the search or the implementation of newly acquired advice. CHIRPI has the potential to help identify parents who are at risk of child-directed cyberchondriac behavior. More research is needed to determine the potentially negative consequences of excessive searching for Web-based information related to one’s children’s health, eg, increased use of health services, children being subjected to unnecessary diagnostic procedures, and the transmission of unsubstantiated health concerns. The Distress subscale of CHIRPI, in particular, seems to be a useful marker of dysfunctional search influences, such as parents’ anxiety about their own health or self-directed cyberchondria. It may indicate general patterns of factors related to anxious health-related cognitions, emotions, and behaviors, which the parents also apply to the health of their children. Hopefully, the availability of CHIRPI will improve our understanding of the consequences of excessive Web-based searching for health information.

### Practical Implications

Primarily, CHIRPI can be used to gain greater knowledge of health-related Web searches by parents and the possible consequences of such search behavior. The authors do not wish to suggest that searching for such information is problematic in itself. However, if it is coupled with health anxiety and cyberchondria regarding the parents themselves, it may become excessive and a source of distress for the parents. We would therefore suggest using this instrument to assess whether a person being treated for health anxiety also searches for their children and, if appropriate, discuss this behavior in the course of the treatment. Future research should address whether parents’ excessive Web searches for health information regarding their children also leads to problematic consequences for the children themselves.

## Appendix

Multimedia Appendix 1 Checklist for Reporting Results of Internet E-Surveys checklist.

Multimedia Appendix 2 CHIRPI scale in German and English.

Multimedia Appendix 3 Independent samples t-tests comparing participants who opted to take part in the retest and those who did not.

Multimedia Appendix 4 Scores for the Children’s Health Internet Research, Parental Inventory (CHIRPI) total score (A), and its subscales (B-D) as a function of the child’s health status and the perceived vulnerability regarding the child’s health assessed using the Child Vulnerability Scale (CVS).

## Abbreviations

ANOVA: analysis of variance
CHIRPI: Children’s Health Internet Research, Parental Inventory
CSS: Cyberchondria Severity Scale
CVS: Child Vulnerability Scale
EFA: exploratory factor analysis
KMO: Kaiser-Meyer-Olkin criterion
MAP: minimum average partial
mSHAI: modified short Health Anxiety Inventory
VAS: visual analog scale
