# Supplementary material for: Development of an Instrument to Assess Parents’ Excessive Web-Based Searches for Information Pertaining to Their Children’s Health: The “Children’s Health Internet Research, Parental Inventory” (CHIRPI)
Source: J Med Internet Res. 2020 Apr 15;22(4):e16148. doi: 10.2196/16148 (PMC7191340; doi:10.2196/16148)
Supplement: Multimedia Appendix 1 [file jmir_v22i4e16148_app1.pdf]

S3 CHERRIES Checklist

Design	Target population: German-speaking parents with at least one child under the age of 10 years<br>Convenience sample
Ethics	
IRB	IRB approval was obtained.
Informed consent	Participants were told:<br>- purpose of the survey<br>- length of the survey<br>- responsible investigator (AB)<br>- other persons who collected data and administered survey under supervision (advanced students)<br>- information regarding data security (in accordance with German legislation)<br>The had to click a dedicated option box registering their consent before they could proceed to the questionnaire
Data protection	Data was collected anonymously.<br>For persons willing to participate in the retest, the data was pseudonymized using a self-generated code to link the data sets.<br>They also provided an email address so we could invite them for the retest. This email was entered into an unrelated system (the main survey was conducted with LimeSurvey and the emails were collected with SurveyMonkey) so that no relationship existed.<br>The data server was built with lime survey and hosted behind the firewall of Göttingen University.<br>All data handling and storage complied with the German data protection legislation.
Development and Pre-testing	The survey was developed using the established survey software Limesurvey to ensure functioning of the survey infrastructure.<br>The study information, consent text and questions were entered, proofread and extensively tested in questionnaire mode in several steps: (1) The researchers themselves tested the questionnaire on several platforms (desktop, laptop, smartphone) and most used browsers for adaptation to screen size, text size, complete display of answering options, etc.<br>(2) The researchers checked whether the recorded variables were correct (correspondence of user entry and value in data matrix).<br>(3) Students and friends with children tested the survey for any questions or ambiguities as well as technical problems.
Recruitment process and description of sample having access to the questionnaire	
Survey mode	The survey was hosted as an open survey. Everyone who saw the link was able to participate.
Contact mode	General web pages, social media groups, snowball system and distribution via the university's general mailer (i.e. including all staff, academic and non-academic).
Advertising	It was widely posted on pages intended for parents and related groups etc. The advertisement was held short and neutral. In addition we put recruitment notes up in nursery schools, supermarkets etc.<br>Original text:

	Liebe Eltern,<br>*vielen Dank für die Aufnahme in Ihre Gruppe!*<br>*Im Rahmen eines Forschungsprojektes der Georg-August-Universität Göttingen und der Philipps-Universität Marburg suchen wir Eltern mit Kindern zwischen 0-10 Jahren für eine Fragebogenuntersuchung. Vielleicht haben Sie ja Interesse, daran teilzunehmen?*<br>*Untersucht wird, wie Eltern im Internet nach Gesundheitsinformationen für ihre Kinder suchen und wie sie diese Informationen nutzen. Leider können Sie den Fragebogen nur ausfüllen, wenn Sie mindestens ein Kind im Alter von 0-10 Jahren haben. Teilnehmen können sowohl Mütter als auch Väter.*<br>*Wir würden uns sehr freuen, wenn Sie sich ca. 15 Minuten Zeit nehmen und uns bei unserer Forschung unterstützen. Als Dank für Ihre Teilnahme können Sie einen von vier ……. Gutscheinen im Wert von je 25€ gewinnen.*<br>*Unter folgendem Link können Sie an der Studie teilnehmen https://link...*<br>*Vielen Dank im Voraus für Ihre Unterstützung!*<br>*Bei Fragen stehen wir Ihnen gerne zur Verfügung: ….*
Survey Administration	
Type of survey	Hosted on webpage.
Context	Differing pages: general health pages, activities for children, clothes and toy swapping, nursery schools etc., children services, snowballing among parents. The university mailing list reaches every staff member from maintenance personnel to professors.
Voluntariness	Voluntary
Randomization of items	Items were not randomized as established questionnaires were used.
Adaptive questioning	In some instances: Number of children – the questions for age and sex of the children were displayed according to the number selected.
Number of items per page	On average between 8-12
Number of screen pages	10 screen pages (excluding Information and consent and final page where participants could use comments).
Completeness check	No completeness checks were done. Mandatory items were those required to ascertain inclusion criteria.
Review step	No explicit review options were implemented.
Response rates	
Unique site visitors	No identifying information such as IP addresses were collected. In total, the page had 6121 visits.
View rate	Not applicable since no unique site visitors were identified
Participation rate	Number of persons who filled in informed consent: 515
Completion rate	353/515 = 68.5%<br>partially completed questionnaires were also included, provided the participant completed CHIRPI. This was the case for 394 participants: 394/515 = 76.5%
Preventing multiple entries from the same individual	
Cookies	Not used

IP check	Not used. Saving IP addresses was deemed problematic for reasons of data protection.
Log file analysis	We checked double entries in terms of same sex / age / number and age / sex of children. None were identified.
Registration	Not applicable
Analysis	
Handling of incomplete questionnaires	All questionnaires that completed at least the CHIRPI were analysed. This included some that broke off afterwards. Parents with small children may easily get called away and there was no reason to exclude them from analysis.
Questionnaires submitted with an atypical time stamp	Not applicable
Statistical correction	None
