# Supplementary material for: Development of an Instrument to Assess Parents’ Excessive Web-Based Searches for Information Pertaining to Their Children’s Health: The “Children’s Health Internet Research, Parental Inventory” (CHIRPI)
Source: J Med Internet Res. 2020 Apr 15;22(4):e16148. doi: 10.2196/16148 (PMC7191340; doi:10.2196/16148)
Supplement: Multimedia Appendix 3 [file jmir_v22i4e16148_app3.docx]

Table S4. Comparison of the retest participants and the other participants: independent-samples *t-*tests on age, number of children, and questionnaire results and chi-squared test of sex distribution.

|  | No Retest (*n* = 288) | | Retest (*n* = 73) | |  |  |  |  |
| --- | --- | --- | --- | --- | --- | --- | --- | --- |
| Variable | Mean | SD | Mean | SD | *df* | *t* | *P* | *d* |
|  |  |  |  |  |  |  |  |  |
| Age | 32.6 | 5.9 | 33.3 | 4.9 | 359 | -0.85 | .393 | -0.12 |
| Number of children | 1.9 | 1.2 | 1.5 | 0.6 | 359 | 2.31* | .022 | 0.35 |
| CHIRPI | 45.3 | 11.6 | 47.6 | 10.7 | 359 | -1.58 | .114 | -0.21 |
| CSS-15 | 29.3 | 29.8 | 8.0 | 7.7 | 352 | 1.90 | .658 | -0.04 |
| mSHAI | 27.8 | 25.0 | 11.2 | 9.8 | 342 | -0.44 | .053 | 0.27 |
| CVS | 5.8 | 5.9 | 4.4 | 3.8 | 359 | -0.31 | .798 | -0.06 |
|  |  |  |  |  |  |  |  |  |
|  | Women | Men | Women | Men |  | χ^2^ |  |  |
| Sex | 302 | 9 | 70 | 3 | 1 | 0.29 | .591 |  |

Notes: CHIRPI: Children’s Health Internet Research Parental Inventory; CSS-15: Cyberchondria Severity Scale, short form; mSHAI: Modified Short Health Anxiety Inventory; CVS: Child Vulnerability Scale. Total number in this table is not the same as the sample size due to exclusions from the retest sample.

Table S5. Correlations between the full scale or subscales and demographic variables, time spent searching the Internet for information related to one’s children’s health, health-related questionnaires and VASs. All *r* and *P* values shown.

| Variables | CHIRPI score |  | Dis |  | SF |  | IA |  |
| --- | --- | --- | --- | --- | --- | --- | --- | --- |
|  | *r* | *P* ^a^ | *r* | *P* ^a^ | *r* | *P* | *r* | *P* |
|  |  |  |  |  |  |  |  |  |
| Age | -.01 | .87642 | -.04 | .47303 | .02 | .69432 | -.03 | .60848 |
| Years of education | .00 | .92748 | -.07 | .16660 | .10 | .04631 | -.12 | .02094 |
| Number of children | -.12 | .02044 | -.09 | .07381 | -.15 | .00257 | .00 | .96187 |
| Mean age of children (years) | -.17 | .00095 | -.06 | .24353 | -.27 | 1.05E-07 | .03 | .56100 |
| Search time, week without symptoms (mins) | .14 | .00693 | .04 | .48822 | .11 | .02823 | .19 | .00022 |
| Search time, week with symptoms (mins) | .26 | 3.68E-07 | .11 | .04012 | .27 | 5.26E-08 | .20 | 6.47E-05 |
| CVS | .29 | 2.45E-08 | .54 | 3.07E-28 | .15 | .00459 | .27 | 2.05E-07 |
| mSHAI | .39 | 5.66E-14 | .50 | 2.66E-23 | .21 | 6.74E-05 | .22 | 2.76E-05 |
| CSS-15 | .66 |  | .61 |  | .51 | 1.03E-23 | .46 | 2.74E-19 |
| VAS worry/anxiety regarding child’s health | .38 | 8.93E-13 | .50 | 7.17E-23 | .22 | 5.35E-05 | .21 | 6.80E-05 |
| VAS child’s health | -.09 | .08467 | -.11 | .02842 | .00 | .96720 | -.09 | .09498 |
| VAS participant’s medical competence | .03 | .64496 | -.08 | .17698 | .02 | .68303 | .14 | .01154 |
| VAS experience with child’s doctors | -.20 | .00022 | -.09 | .10959 | -.19 | .00075 | -.19 | .00044 |

Notes: ^a^ In the empty cells the P was too small for calculation with SPSS. The threshold for Bonferroni corrections is *P*<.00096.

CHIRPI: Children’s Health Internet Research Parental Inventory; Dis: Subscale Distress; SF: Subscale Symptom Focus; IA: Subscale Implementing Advice; CVS: Child Vulnerability Scale; mSHAI: Modified Health Anxiety Inventory; CSS-15: Cyberchondria Severity Scale (15-item version); VAS: visual analogue scale (0‑100; 0 signifying, respectively, no worries about child’s health, child’s health is poor, parent has no medical competence, parent’s experience with child’s doctors has been poor).
