# Supplementary figures and images for: Development of an Instrument to Assess Parents’ Excessive Web-Based Searches for Information Pertaining to Their Children’s Health: The “Children’s Health Internet Research, Parental Inventory” (CHIRPI)
Source: J Med Internet Res. 2020 Apr 15;22(4):e16148. doi: 10.2196/16148 (PMC7191340; doi:10.2196/16148)

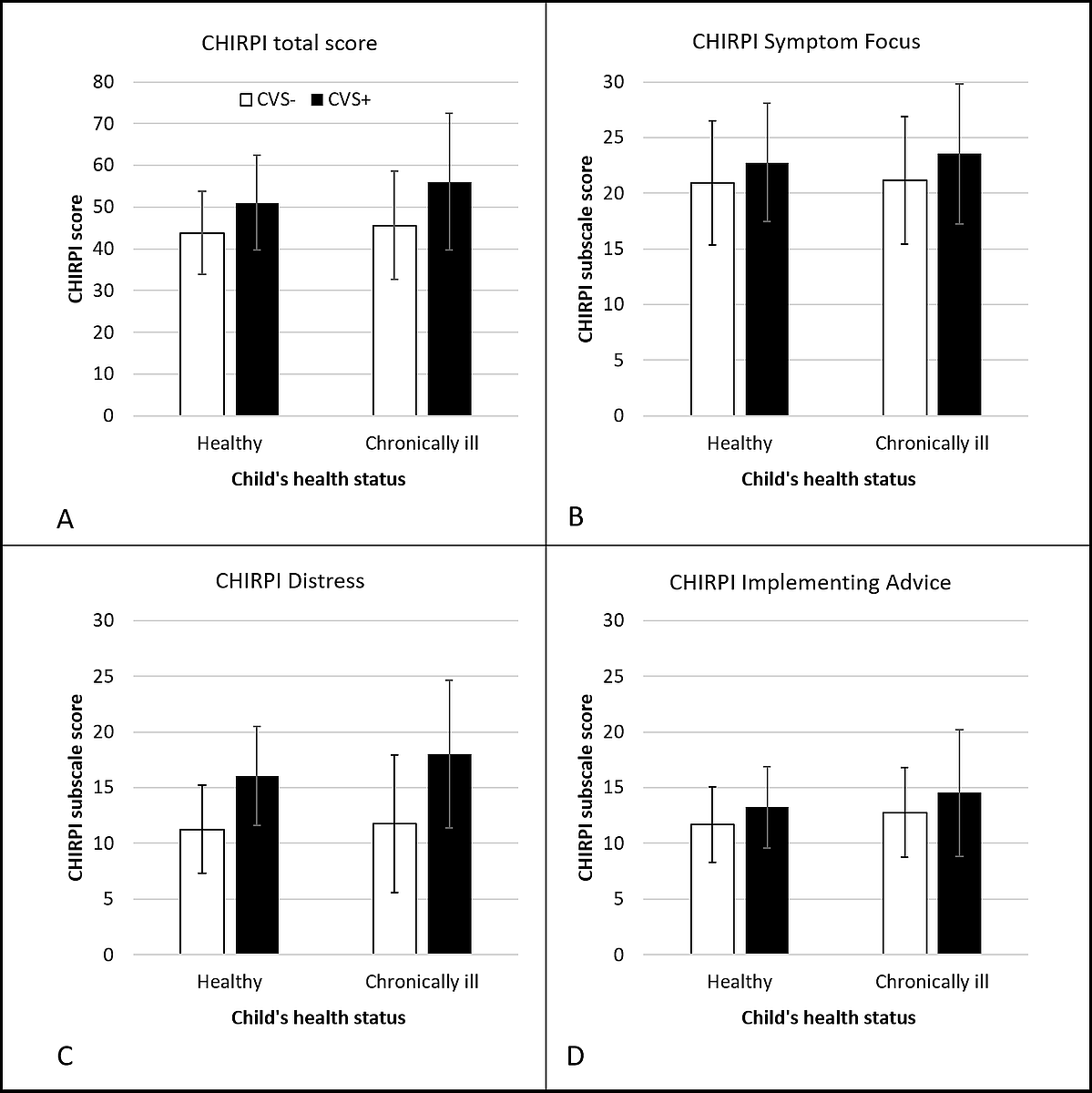

Supplement: Multimedia Appendix 4 [file jmir_v22i4e16148_app4.png]
